# Genetic considerations for mollusk production in aquaculture: current state of knowledge

**DOI:** 10.3389/fgene.2014.00435

**Published:** 2014-12-10

**Authors:** Marcela P. Astorga

**Affiliations:** Instituto de Acuicultura, Universidad Austral de ChilePuerto Montt, Chile

**Keywords:** mollusk, genomic, aquaculture, genetic improvement, genetic, bivalves

## Abstract

In 2012, world mollusk production in aquaculture reached a volume of 15,171,000 tons, representing 23% of total aquaculture production and positioning mollusks as the second most important category of aquaculture products (fishes are the first). Clams and oysters are the mollusk species with the highest production levels, followed in descending order by mussels, scallops, and abalones. In view of the increasing importance attached to genetic information on aquaculture, which can help with good maintenance and thus the sustainability of production, the present work offers a review of the state of knowledge on genetic and genomic information about mollusks produced in aquaculture. The analysis was applied to mollusks which are of importance for aquaculture, with emphasis on the 5 species with the highest production levels. According to FAO, these are: Japanese clam *Ruditapes philippinarum*; Pacific oyster *Crassostrea gigas*; Chilean mussel *Mytilus chilensis*; Blood clam *Anadara granosa* and Chinese clam *Sinonovacula constricta*. To date, the genomes of 5 species of mollusks have been sequenced, only one of which, *Crassostrea gigas*, coincides with the species with the greatest production in aquaculture. Another important species whose genome has been sequenced is *Mytilus galloprovincialis*, which is the second most important mussel in aquaculture production, after *M. chilensis*. Few genetic improvement programs have been reported in comparison with the number reported in fish species. The most commonly investigated species are oysters, with at least 5 genetic improvement programs reported, followed by abalones with 2 programs and mussels with one. The results of this work will establish the current situation with respect to the genetics of mollusks which are of importance for aquaculture production, in order to assist future decisions to ensure the sustainability of these resources.

## Aquaculture

The aquaculture industry consists of 3 main groups with different production volumes: fish, with a volume of 44.15 million tons in 2012, representing 66.3% of production; mollusks with 15.17 million tons equivalent to 22.8%; crustaceans, 6.44 million tons, 9.7%. The production of other types was 0.86 million tons, or 1.3% (FAO, 2014). Based on these values, mollusks form the second largest group; however the levels of biotechnological research and the technologies applied are very low compared to the high level of development in cultivated fish species.

Biotechnology and genetic research have developed strongly in the last decade, and a large database now exists for fish species of importance for aquaculture (NCBI, Genbank, EMBL). These new research lines have permitted great progress and increased efficiency. Research of this kind has led to the development of many applications for fish production, with an increase in the number of techniques for genetic improvement, and a wider range of genetic variability of the resources for application in studies of traceability, pedigree, degrees of relatedness, and the search for molecular markers for Marked Assisted Selection. These developments are well reviewed in Hulata (2001), Liu and Cordes (2004), and Martínez (2007); genetic improvement programs are reviewed in Gjedrem (2012) and Gjedrem et al. (2012). In mollusk production, however, the development of such knowledge has been minimal (for Latin American mollusks see Astorga, 2008).

The present work therefore reviews the current state of scientific knowledge on the genetics of mollusk species which are important for aquaculture. The amount of existing information on the genomes of these resources will be assessed—this is needed to establish a baseline for knowledge-based decision-making. Such knowledge would also enable us to define brood-stock selection programs, generate the bases for genetic improvement programs and finally assess the state of a given resource in order to ensure its long term conservation and sustainability.

## Mollusk production

World mollusk production in 2012 was 15.17 million tons. Total aquaculture production of mollusks is represented by 5 main groups: clams, oysters, mussels, scallops, and abalones. Clam production during 2011 was 4.929 million tons; this was followed by oysters with 4.519 million tons; mussels with 1.802 million tons; scallops with 1.52 million tons; and finally abalones with aquaculture production of 395,000 tons (FAO, 2013).

There are 5 principal mollusk species produced in aquaculture (i.e., with production of over 250,000 tons), all bivalves, which correspond to only 5 species of mollusks reported in the FAO statistics (2013). Three of these are clams: *Ruditapes philippinarum* or Japanese clam; *Anadara granosa (=Tegillarca granosa)*, Blood clam or Blood cockle from Malaysia; and *Sinonovacula constricta* or Chinese clam. The other two are the oyster *Crassostrea gigas* and the Chilean mussel *Mytilus chilensis*. The detailed production for each of these is shown in Table 1, including the economic value generated by the production of these 5 resources with the highest volume in shellfish aquaculture. In this review the state of knowledge on the genetics of each of these 5 species will be evaluated.

**Table 1:** **Aquaculture production and economic value of the 5 mollusk species with production greater than 250,000 tons (FAO, 2013), with review of genetic and genome information**.

**Species**	**Common**	**Production**	**Total**	**WebScience**	**PubMed**	**Sequences**	**EST**	**Protein**
	**name**	**‘000 Ton**.	**value MUS$**	**publications**	**publications**	**in GenBank**	**in NCBI**	**in NCBI**
*Ruditapes philippinarum*	Japanese clam	3681	3438	25	273	30,442	6,469	1046
*Crassostrea gigas*	Pacific oyster	637	1323	200	1821	11,775	206,647	29,004
*Mytilus chilensis*	Chilean mussel	288	1148	15	35	119	7	27
*Anadara granosa*	Blood clam	393	468	6	48	641	2,278	158
*Sinonovacula constricta*	Chinese clam	744	670	5	23	16,383	5,296	204

The genetic information is based on the number of genetics-related publications in Web of Science databases, NCBI, PubMed, databases in GenBank, EST (Expressed Sequences Tag) regions and coded proteins.

## Genetic resources

Genetic resources is the name given to all the genetic or genome information for a group of representative individuals of a species which constitutes a natural resource (CBD Nagoya Protocol, 2010). Knowledge of the genetic resources of species has become increasingly important with the discovery of the importance of genetic indicators for species conservation, obtaining sufficient raw material to generate successful improvement programs, the sustainability of natural banks and the appropriate management of genetic resources (Kenchington et al., 2003).

Correct use of genetic resources allows the species to be developed sustainably, maintaining it for future generations; this information is also the basis for new genetic improvement programs in species in early or advanced stages of aquaculture (Kenchington et al., 2003; Guo, 2009; Lind et al., 2012). To start mollusk production in a hatchery, Gaffney (2006) proposes that high levels of genetic diversity must be ensured among the brood-stock. Proper levels of variability must then be maintained over time, by monitoring genetic variability to avoid excessive inbreeding (Gaffney, 2006). These factors—which require the application of biotechnologies or the design of genetic improvement plans—have been identified as important for starting aquaculture production.

Genetic improvement programs require reproductive management throughout the whole cycle of the species. Mollusk production is still carried out in extensive aquaculture facilities, where the initial production stages depend completely on the natural environment and on seed obtained from natural banks for the fattening stage, which makes it difficult to design breeding programs. On the other hand, the costs of obtaining seed from the natural environment are drastically lower than those of seed production in a hatchery, threatening the profitability of mollusk production companies and their ability to invest in genetic improvement programs. These programs may become very profitable, but only if the sale value of the product is very high, as occurs with the oyster, or if the genetic gain is directly related with a reduction in production costs.

## Genetic information on the main aquaculture species

(Sort by economic value of production according to Table 1).

### Ruditapes philippinarum

**Figure 1:**
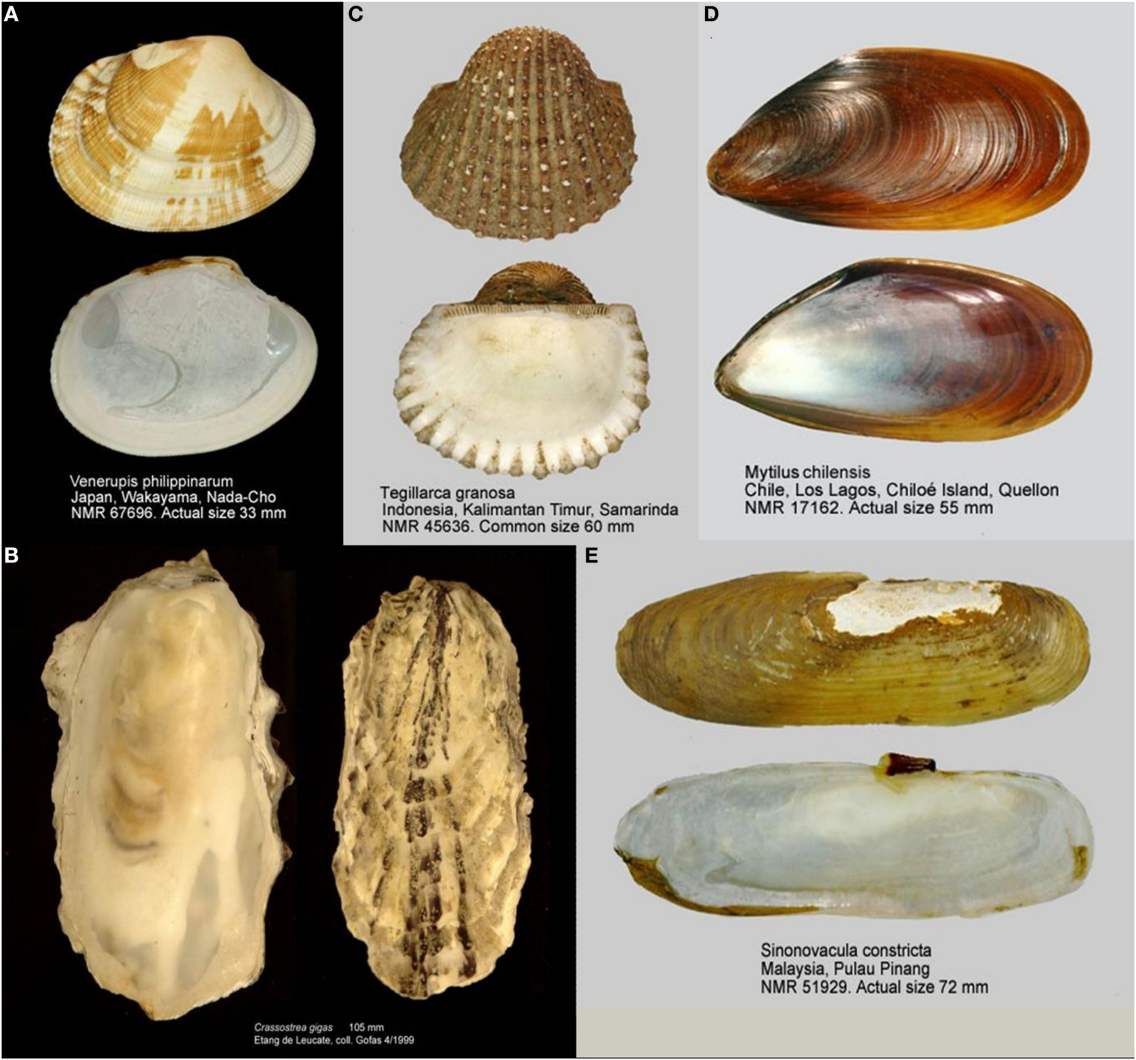
**Photograph of the 5 mollusk species with the greatest production in aquaculture**. **(A)** *Ruditapes philippinarum* or *Venerupis philippinarum*. **(B)** *Crassotrea gigas*. **(C)** *Mytilus chilensis*. **(D)** *Tegillarca granosa* or *Anadara granosa*. **(E)** *Sinonovacula constricta*. All images extracted from WoRMS (www.marinespeciers.org) with use authorized. Images **(A,C–E)** by Joop Trausel and Frans Slieker, Author: Natural History Museum Rotterdam. Image **(B)** by Serge Gofras, Muséum national d'histoire naturelle (MNHN).

Genetic information on the Malaysian clam *Ruditapes philippinarum* (Figure 1A), also known as *Venerupis philippinarum*, was searched using the ISI Web of Science and the National Center of Biotechnology Information (NCBI) search engines and the PubMed database. This allowed us to establish the low level of existing genetic information, despite the fact that it is one of the 5 mollusk species with the highest production in the world and is located in the first place of economic value production. Only around 30 papers discussing the genetics of this species were found (see database at Annex 1), although we consider that the number is not always representative of the information available. The research focused mainly on the genetic characterization of populations and the evaluation of genetic differentiation and population structure (42%), followed by searches for molecular markers (17%), and evaluation of hybrids with congeneric species (13%). A few papers describe research related to the genetic response associated with the immune system, gene expression, and transcriptome (total 21%), and finally 2 works address heritability and selection measurement (8%). However, although the main focus of research is on molecular genetics and molecular biology, a large number of works was found associated with study of gene expression as it relates to the immune response or to exposure to contaminants; there were at least 19 papers from 2012 to date, showing a strong focus on the transcriptome of this species. On searching for genome information in the National Center for Biotechnology Information (NCBI), data was found on 30,442 DNA, and RNA sequences, 6469 EST (Expressed Sequence Tag) sequences, and 1046 protein sequences (Table 1). Little genetic information is available on this species, despite the fact that it has been cultivated in hatcheries since the mid 1970s (Zhang and Yan, 2006). The information is insufficient to generate a basis for developing genetic improvement programs. The heritability of shell length has been calculated in the natural and cultured population at the larval and juvenile stages (Yan et al., 2014); the values found were 0.22 for larvae and 0.39 for juveniles in natural populations, and 0.17 and 0.87 for cultured populations. These results suggest that selection should be highly effective in this species, and that selecting either a natural or a cultured population to obtain faster growth should be successful. Zhao et al. (2012) established that divergent selection is effective and promotes shell length changes in the Manila clam, finding relatively moderate heritability values of 0.165 and 0.260, but quite high genetic gain values, with an increase in growth of 3.55% over the control line. Zhao et al. (2012) found that a base population with high genetic variability should be established as the basis for a divergent selection program. This would allow stable production of high quality seed to ensure the sustainability of aquaculture of this resource.

### Crassostrea gigas

Unlike the species described above, a large quantity of genetic research has been done in the oyster *Crassostrea gigas* (Figure 1B), associated with the search for molecular markers and the genetic characterization of populations. In the area with potential for application in aquaculture, works were found associated with selection response, heritability measurement, and searches for QTL and chromosome manipulation, highlighting the work of Guo et al. (2012), Ge et al. (2014) and Zhong et al. (2014). There were also many works associated with gene expression in relation to environmental stressors and transcriptome analysis (citations from 2010 to date in Annex 2). This agrees with the large amount of information found in the NCBI database on EST (206,647) as compared to the other species with high aquaculture production (Table 1). In general databases of scientific publications, at least 450 citations from the last 10 years were found relating to the genetics of this species. It is therefore one of the bivalve mollusks with the most highly developed genetic and biotechnological research. Furthermore, the nuclear and mitochondrial genomes of this species have been sequenced, which is the case with only 5 species of mollusk and 2 species of bivalve mollusk. There is a specialized database on this oyster, called Gigasbase, run by the IFREMER group in France. At least five genetic improvement programs have been developed for this species (Rye et al., 2010) although Gjedrem et al. (2012) mention only 3 selective breeding programs. There is a large volume of information for the implementation of new genetic improvement programs in the species. For example, we found a work assessing the heritability of shell pigmentation (Evans et al., 2009) which reported high values for narrow sense heritability (*h*^2^ = 0.59). This suggests that total left-shell pigmentation in *C. gigas* is strongly influenced by additive genetic variation and therefore amenable to selection. Another characteristic which has been extensively studied in oysters is the genetic response to the high summer mortality observed in hatcheries (Dégremont et al., 2010). High realized heritabilities (*h*^2^r) values have been estimated for high survival groups (above 0.88), higher than the values for low survival groups (0.55–0.68), and the authors conclude that these values would permit good results in a selective breeding program to improve survival (Dégremont et al., 2010). We also found estimates of growth heritability, with high values reported by Li et al. (2011) with 0.40; 0.33; and 0.15 and Wang et al. (2012)—who also report high heritability values for growth rate (0.457; 0.312; and 0.332), which has allowed a solid basis to be established for the further development of genetic improvement programs for this species, ensuring greater genetic gain in the short term.

### Mytilus chilensis

There is less scientific information published on the mussel *Mytilus chilensis* (Figure 1C) than the two previous species; in August 2014, only 15 papers on the genetics of this species could be found in the Web of Science (Annex 3). Aquaculture production is higher for this species than for other mussels, yet the amount of scientific information published is smaller—for example, around 100 scientific publications were found in the same database for the mussel *Mytilus galloprovincialis*. The largest proportion (50%) of the research published on *M. chilensis* is associated with the genetic characterization and population structure of this species distributed along the Chilean coast. Works can be found on the identification of molecular markers, heritability, selection response and gene expression, all in much the same proportions and small quantities. This is corroborated by a search for genome information in the NCBI databases, where only 119 data were found on sequenced genes, and only 7 EST were identified (Table 1). Of the 5 top species in aquaculture production, *M. chilensis* presents the least amount of genome information and its genome has not been sequenced. However, it should be noted that the genome of the Mytilid species *Mytilus galloprovincialis*, has been sequenced; this species, cultivated principally around the coast of Spain, presents the next highest aquaculture production among mussels.

There is one record of a genetic improvement program for mussels (Rye et al., 2010; Gjedrem et al., 2012); however, it is not being applied in production because cultivation is based on seed obtained from the natural environment and not from hatcheries. Nevertheless, works exist which estimate heritability and response to selection in this species. High heritability values have been found for a variety of characteristics such as growth in shell height and live weight (0.2–0.9) (Toro et al., 2004a). In larvae these parameters present a heritability range between 0.38 and 0.84 (Toro et al., 2004b). Finally, Alcapan et al. (2007) assessed the effects of environment and ageing on the heritability of body size in *M. chilensis*; they observed great variability in heritability values, conditioned by the site and the age of the individuals. These data would allow good results to be obtained in improvement programs, however the high production costs of seed obtained from hatcheries as opposed to the natural environment remains a limiting factor for this species. This makes the implementation of these improvement programs uncompetitive for producers in the short term. Nevertheless, the possibility cannot be ruled out that it may become necessary in future to make seed of high genetic quality available to this production sector.

### Anadara granosa

In the case of the Blood cockle *Anadara granosa* or *Tegillarca granosa* (Figure 1D), at least 4 publications were found on genetic characterization of populations, and two more relating to the search for molecular markers (Annex 4). There is therefore very little genetic information published for this species, despite its high production value. However, when we searched for genome information, we found more research into EST regions in this species (2278) (Table 1) than in *Mytilus chilensis* (only 7 EST). No records were found of current genetic improvement programs in this species, nor any information on baseline data which would allow the development potential of such programs to be assessed. On the other hand, the natural population structure has been studied (Ni et al., 2012; Wang et al., 2013) and this is a pre-requirement for selecting brood-stock and forming families for an improvement program. Wang et al. (2013) reported high genetic variability values, meaning that high diversity exists for brood-stock selection. However, they found genetic divergence in various sites on the coast of China presenting lower genetic variability, possibly caused by the admixture of artificially produced seed. This type of information suggests that genetic improvement programs need to include cross-breeding in their design in order to prevent genetic variability from being reduced, perhaps by using a large number of breeding individuals or a group which is representative of the genetic variability of the population. Ni et al. (2012) observe high genetic structuring in populations, indicating a high risk of erosion and possible local extinction. Populations need to be protected to prevent loss of genetic variability in the species.

### Sinonovacula constricta

Of the 5 top species in aquaculture production, the one with the least scientific information published on genetics is the Chinese clam *Sinonovacula constricta* (Figure 1E). In the Web of Science databases to August 2014, only three publications were found on searches for molecular markers plus two on the genetic evaluation of populations (Annex 5) (Table 1). However, there is a large number of sequenced genes in the NCBI databases, many more than for some other species analyzed. In this respect it ranks third among the top 5 species (Table 1), with the highest number of genes sequenced among the main 5 species produced in aquaculture (16,383 genes). There are no records of genetic improvement programs in this species, nor any publications of estimators for the implementation of such programs. Very little is known about its population structure. Niu et al. (2012) observed high genetic variability in 10 populations on the Chinese coast; however, they found genetic differentiation between sites, and propose the possible presence of cryptic species. This aspect should be studied since it is important to identify the cultivated species correctly and establish different management methods if more than one species exists.

## Concluding remarks

On the basis of this review it is apparent that there is no obvious relationship between the economic importance of a species, in terms of the volume produced in aquaculture, and the amount of scientific information available about genetics/genomic resources, when restricted to the five most produced species. In this case our analysis was carried out for research into genetics, which is the basis for genetic improvement programs in aquaculture species. The scientific information found is summarized, with the databases used, in Table 1. From this we see that the most studied species, using all the genetic information indicators, is *Crassotrea gigas*. There is great interest in this species and it has been introduced into many parts of the world for cultivation. Among mussels, the small amount of genetic information available on *Mytilus chilensis* is striking. At the same time the congeneric species *Mytilus galloprovincialis* is the most studied mussel; furthermore the latter is one of only two mussel species whose genome has been sequenced, and there are 41,294 records of DNA sequences, 19,756 of EST and 2,424 coded proteins. The species with the largest number of DNA sequences is the oyster *Crassostrea angulata*, with 81,015 DNA sequences, even more than *C. gigas*. On the other hand, *C. gigas* is the bivalve mollusk with the most EST sequences identified.

The quantity of genetic information available on bivalve mollusks varies widely, with great differences between species, even though their production volumes in aquaculture are similar. There is also still little information with which to generate solid bases for genetic improvement programs such as are observed in fish or other cultivated species. The only exception is *C. gigas*, for which adequate base information exists and genetic improvement programs are currently being implemented. Sufficient information exists on the mussel *M. chilensis* to provide a basis for more improvement programs, but the low use of reproduction in controlled environments is a limiting factor in this species. For the other three species there is virtually no basic genetic information on which predicting their response to selection programs. Further research is required to generate a sufficient basis for selective reproduction, and also to clarify information on natural populations, identifying possible cryptic species or strong differentiation between populations. The lack of knowledge about species with high production in aquaculture could lead to population bottlenecks, since if the brood-stock management plan is unsuitable, or seed extraction from local populations is not handled correctly, the populations which sustain these resources could be drastically reduced. This could result in a severe diminution of genetic diversity, to the point where it might be difficult for the species to recover, meaning that in future these resources which are so important for aquaculture would not be sustainable.

### Conflict of interest statement

The author declares that the research was conducted in the absence of any commercial or financial relationships that could be construed as a potential conflict of interest.
