# Supplementary material for: Genetic considerations for mollusk production in aquaculture: current state of knowledge
Source: Front Genet. 2014 Dec 10;5:435. doi: 10.3389/fgene.2014.00435 (PMC4261805; doi:10.3389/fgene.2014.00435)
Supplement: Supplementary file 1 [file DataSheet1.DOC]

**Annex 1.**

**References *Ruditapes philippinarum***

Order by publication date, newest to oldest

Milani, L., Ghiselli, F., Iannello, M., and Passamonti, M. (2014). Evidence for somatic transcription of male-transmitted mitochondrial genome in the DUI species *Ruditapes philippinarum* (Bivalvia: Veneridae). *Current Genetics*. 60: 3, 163-173.

Hu, G., Yan, X., Zhu, D., and Nie, H. (2014). Isolation and characterization of fourteen polymorphic microsatellite loci in the Manila clam (*Ruditapes philippinarum*). *Conservation genetics*. 6: 2, 251-253.

Nie, H., Zhu, D., Yang, F., Zhao, L., and Yan, X. (2014). Development and characterization of EST-derived microsatellite makers for Manila clam (*Ruditapes philippinarum*). *Conservation Genetics*. 6: 1, 25-27.

Yan, X., Huo, Z., Yang, F., and Zhang, G. (2014). Heritability of larval and juvenile growth for two stocks of Manila clam *Ruditapes philippinaru. Aquaculture*. 45: 3, 484-490.

Xing, K., Gao, M., and Li, H. (2014). Genetic differentiation between natural and hatchery populations of Manila clam (*Ruditapes philippinarum*) based on microsatellite markers. *Genetic and molecular*. 13: 1, 237-245.

Kitada, S., Fijake, C., Asakura, Y., Yuki, H., Nakajima, K., Vargas, K., Kawashima, S., Hamasaki, S., and Kishino, H. (2013). Molecular and morphological evidence of hybridization between native *Ruditapes philippinarum* and the introduced *Ruditapes* form in Japan. *Conservation Genetics*. 14: 3, 717-733.

An, H., Park, K., Cho, K., Han, H., and Myeong, J. (2012). Genetic structure of Korean populations of the clam *Ruditapes philippinarum* inferred from microsatellite marker analysis. *Biochemical systematics and ecology*. 44. 186-195.

Mao, Y., Gao, T., Yanagimoto, T., and Xiao, Y. (2011). Molecular phylogeography of *Ruditapes philippinarum* in the Northwestern Pacific Ocean based on COI gene. *Journal of experimental marine biology and ecology*. 407: 2, 171-181.

Hurtado, N., Perez-Garcia, C., Moran, P., and Pasantes Juan, J. (2011). Genetic and cytological evidence of hybridization between native Ruditapes decussatus and introduced *Ruditapes philippinarum* (Mollusca, Bivalvia, Veneridae) in NW Spain. *Aquaculture*. 311: 1-4, 123-128.

Poulain, C., Lorrain, A., Flye-Sainte-Marie, J., Amice, E., Morize, E., and Paulet, Y-M. (2011). An environmentally induced tidal periodicity of microgrowth increment formation in subtidal populations of the clam *Ruditapes philippinaru. Journal of experimental marine biology and ecology.* 397: 1. 58-64.

An, H., Kim, E., and Park, Y. (2009). Isolation and characterization of microsatellite markers for the clam *Ruditapes philippinarum* and cross-species amplification with the clam *Ruditapes variegat. Conservation genetics*. 10: 6, 1821-1823.

Liu, X., Bao, Z., Hu, J., Wang, S., Zhan, A., Liu, H., Fang, J., and Wang J. (2007). AFLP analysis revealed differences in genetic diversity of four natural populations of Manila clam (*Ruditapes philippinarum*) in China. *Acta oceanologica sinica*. 26: 1, 150-158.

Yasuda, N., Nagai, S., Yamaguchi, S., Lian, C., and Hamaguchi, M. (2007). Development of microsatellite markers for the Manila clam *Ruditapes philippinarum. Molecular ecology notes.* 7: 1, 43-45.

Park, GM., Chung, EY., and Hur, SB. (2002). Genetic characters of the Korean shortnecked clam *Ruditapes philippinarum* based on ITS2 and mtCOI gene sequences. *Korean journal of genetics.* 24: 1, 51-58.

Fernández, A., García, T., Asensio, L., Rodríguez, MA., González, I., Céspedes, A., Hernández, PE., and Martín, R. (2000). Identification of the clam species Ruditapes decussatus (grooved carpet shell), Venerupis pullastra (pullet carpet shell), and *Ruditapes* *philippinarum* (Japanese carpet shell) by PCR-RFLP. *Journal of agricultural and food chemistry.* 48: 8, 3336-3341.

Zhao, L., Yan, X., Huo, Z., Yang, F., and Zhang, G. (2012). Divergent Selection for Shell Length in the Manila Clam, *Ruditapes philippinarum. Journal of the world aquaculture society.* 43: 6, 878-884.

Moreira, R., Balseiro, P., Planas, J., Fuste, B., Beltran, S., Novoa, B., and Figueras, A. (2012). Transcriptomics of In Vitro Immune-Stimulated Hemocytes from the Manila Clam *Ruditapes philippinarum* Using High-Throughput Sequencing. *Plos ONE.* 7: 4.

Mura, L., Cossu, P., Cannas, A., Scarpa, F., Sanna, D., Dedola, G., Floris, R., Lai, T., Cristo, B., Curini-Galleti, M., Fois, N., and Casu, M. (2012). Genetic variability in the Sardinian population of the manila clam, *Ruditapes philippinaru. Biochemical systematics ecology.* 42, 74-82.

Wang, Q., Wu, H., Zhang, L., and Zhao, J. Defensin B gene for *Ruditapes philippinarum*, comprises nucleotide sequence. *Chinese acad sci yantai inst coastal zon.*

Chiesa, S., Marzano, F., Minervini, G., De Lucrezia, D., Baccarani, G.m Bordignon, G., Poli, I., Ravagnan, G., and Argese, E. (2011). The invasive Manila clam *Ruditapes* *philippinarum* (Adams and Reeve, 1850) in Northern Adriatic Sea: Population genetics assessed by an integrated molecular approach. *Fisheries research.* 110: 2, 259-267.

Vargas, K., Asakura, Y., Ikeda. M., Taniguchi, N., Obata, Y., Hamasaki, K., Tsuchiya, S., and Kitada S. (2008). Allozyme variation of littleneck clam *Ruditapes philippinarum* and genetic mixture analysis of foreign clams in Ariake Sea and Shiranui Sea off Kyushu Island, Japan. *Fisheries Science.* 74: 3, 533-543.

Moraga, D., Mdelgi-Lasram, E., Romdhane, MS., El Abed, A., Boutet, I., Tanguy, A., and Auffret, M. (2002). Genetic responses to metal contamination in two clams: *Ruditapes decussatus* and *Ruditapes philippinaru. Marine environmental research.* 54: 3-5, 521-525.

Peignon, JM., Gerard, A., Naciri, Y., Ledu, C., and Phelipot, P. (1995). Analysis of shell color determinism in the manila clam *Ruditapes-philippinarum. Aquatic living resources.* 8: 2, 181-189.
