# Supplementary material for: Genetic considerations for mollusk production in aquaculture: current state of knowledge
Source: Front Genet. 2014 Dec 10;5:435. doi: 10.3389/fgene.2014.00435 (PMC4261805; doi:10.3389/fgene.2014.00435)
Supplement: Supplementary file 2 [file DataSheet2.DOC]

**Annex 2.**

**References *Crassotrea gigas* 2010-2014.**

**Source: Web of Science.**

Order by publication date, newest to oldest

An, H. S., Kim, W. J., Lim, H. J., Byun, S. G., Hur, Y. B., Park, J. Y., Myeong, J. I. A., and Chul M. (2014). Genetic structure and diversity of *Crassostrea* *gigas* in Korea revealed from microsatellite markers. *Biochemical Systematics and Ecology*. 55, 283-291.

Cong, R., Kong, L., Yu, H., and Li, Q. (2014). Association between polymorphism in the insulin receptor-related receptor gene and growth traits in the Pacific oyster *Crassostrea* *gigas*. *Biochemical Systematics and Ecology*. 54, 144-149.

Zhang, Z., Wang, X., Zhang, Q., and Allen, S. Jr. (2014). Preferential bivalent formation in tetraploid male of Pacific oyster *Crassostrea* *gigas* Thunberg. *Journal of Ocean University of China*. 13: 2, 297-302.

Zhang, Z., Wang, X., Zhang, Q., and Allen, S. Jr. (2014). Cytogenetic mechanism for the aneuploidy and mosaicism found in tetraploid Pacific oyster *Crassostrea* *gigas* (Thunberg). *Journal of Ocean University of China*. 13: 1, 125-131.

Kong, L., Bai, J., and Li, Qi. (2013). Comparative assessment of genomic SSR, EST-SSR and EST-SNP markers for evaluation of the genetic diversity of wild and cultured Pacific oyster, *Crassostrea* *gigas* Thunberg. *Aquaculture*. 420, S85-S91.

Wang, J., Qi, H., Li, L., and Zhang, G. (2014). Genome-wide survey and analysis of microsatellites in the Pacific oyster genome: abundance, distribution, and potential for marker development. *Chinese Journal of Oceanology and Limnology*. 32: 1, 8-21.

An, H. S., Lee, J.W., Kim, W. J., Lim, H. J., Kim, E. M. Byun, S. G., Hur, Y. B., Park, J. Y., Myeong, J. I., and An, C. M. (2013).Comparative genetic diversity of wild and hatchery-produced Pacific oyster (*Crassostrea* *gigas*) populations in Korea using multiplex PCR assays with nine polymorphic microsatellite markers. *Genes & Genomics*. 35: 6, 805-815.

Rohfritsch, A., Bierne, N., Boudry, P., Heurtebise, S., Cornette, F., and Lapegue, S. (2013). Population genomics shed light on the demographic and adaptive histories of European invasion in the Pacific oyster, *Crassostrea* *gigas*. *Evolutionary Applications*. 6: 7, 1064-1078.

Jiang, Q., Li, Q., Yu, H., and Kong, L.-F. (2013). Genetic and epigenetic variation in mass selection populations of Pacific oyster *Crassostrea* *gigas*. *Genes & Genomics*. 35: 5, 641-647

Mat, A. M., Haberkorn, H., Bourdineaud, J.-P., Massabuau, J.-Ch., and Tran, D. (2013).Genetic and genotoxic impacts in the oyster *Crassostrea* *gigas* exposed to the harmful alga *Alexandrium minutum*. *Aquatic Toxicology*. 140, 458-465.

Zhong, X., Li, Q., Yu, H., and Kong, L. (2013). Development and Validation of Single-nucleotide Polymorphism Markers in the Pacific Oyster, *Crassostrea* *gigas*, Using High-resolution Melting Analysis. *Journal of the World Aquaculture Society* 44: 3, 455-465.

Cong, R., Li, Q., and Kong, L. (2013) Polymorphism in the insulin-related peptide gene and its association with growth traits in the Pacific oyster *Crassostrea* *gigas*. *Biochemical Systematics and Ecology*. 46, 36-43.

Meistertzheim, A.-L., Arnaud-Haond, S., Boudry, P., and Thebault, M.-T. (2013). Genetic structure of wild European populations of the invasive Pacific oyster *Crassostrea* *gigas* due to aquaculture practices *Marine Biology*. 160: 2, 453- 463.

Kochmann, J., Carlsson, J., Crowe, T. P., and Mariani, S. (2012). Genetic Evidence for the Uncoupling of Local Aquaculture Activities and a Population of an Invasive Species-A Case Study of Pacific Oysters (*Crassostrea* *gigas*). *Journal of Heredity*. 103: 5, 661-671

Rosa, R.D., de Lorgeril, J., Tailliez, P., Bruno, R., Piquemal, D., and Bachere, E. (2012). A hemocyte gene expression signature correlated with predictive capacity of oysters to survive Vibrio infections. *BMC Genomics*. 13, 252

Sekino, M., Sato, S. Hong, J.-S., and Li, Q. (2012). Contrasting pattern of mitochondrial population diversity between an estuarine bivalve, the Kumamoto oyster *Crassostrea* *sikamea*, and the closely related Pacific oyster *C*. *gigas*. *Marine Biology*, 159: 12, 2757-2776.

Guo, X. Li, Q. Wang, Q.Z., and Kong, L.F. (2012). Genetic Mapping and QTL Analysis of Growth-Related Traits in the Pacific Oyster. *Marine Biotechnology*. 14: 2, 218-226.

Wang, Q., Li, Q., Kong, L., and Yu, R. (2012). Response to selection for fast growth in the second generation of Pacific oyster (*Crassostrea* *gigas*). *Journal of Ocean University of China*. 11: 3, 413-418.

Miller, P.A., Elliott, N.G., Koutoulis, A., Kube, P.D., and Vaillancourt, R.E. (2012). Genetic Diversity Of Cultured, Naturalized, And Native Pacific Oysters, *Crassostrea* *Gigas*, Determined From Multiplexed Microsatellite Markers. *Journal of Shellfish Research*. 31: 3, 611-617 .

Plough, L.V., (2012). Environmental stress increases selection against and dominance of deleterious mutations in inbred families of the Pacific oyster *Crassostrea* *gigas*. *Molecular Ecology*. 21: 16, 3974-3987.

David, E., Tanguy, A., and Moraga, D. (2012). Characterisation and genetic polymorphism of metallothionein gene CgMT4 in experimental families of Pacific oyster *Crassostrea* *gigas* displaying summer mortality. *Biomarkers*, 17: 1, 85-95.

Zhang, Y., Wang, Z., Yan, X., Yu, R., Kong, J., Liu, J., Li, X., Li, Y., and Guo, X. (2012). Laboratory Hybridization Between Two Oysters: *Crassostrea* *Gigas* And *Crassostrea* Hongkongensis. *Journal Of Shellfish Research*. 31: 3, 619-625.

David, E., Tanguy, A., Riso, R., Quiniou, L., Laroche, J., and Moraga, D. (2012). Responses of Pacific oyster *Crassostrea* *gigas* populations to abiotic stress in environmentally contrasted estuaries along the Atlantic coast of France. *Aquatic Toxicology*. 109, 70-79.

Li, Q., Wang, Q., Liu, S., and Kong, L. (2011). Selection response and realized heritability for growth in three stocks of the Pacific oyster *Crassostrea* *gigas*. *Fisheries Science*. 77: 4, 643-648.

Dheilly, N.M., Lelong, C., Huvet, A., Kellner, K., Dubos, M.-P., Riviere, G., Boudry, P., and Favrel, P. (2011). Gametogenesis in the Pacific Oyster *Crassostrea* *gigas*: A Microarrays-Based Analysis Identifies Sex and Stage Specific Genes. *Plos One*. 7: 5,

Plough, L.V., and Hedgecock, D. (2011). Quantitative Trait Locus Analysis of Stage-Specific Inbreeding Depression in the Pacific Oyster *Crassostrea* *gigas*. *Genetics*, 189: 4, 1473-

de Lorgeril, J., Zenagui, R., Rosa, R.D., Piquemal, D., and Bachere, E. (2011). Whole Transcriptome Profiling of Successful Immune Response to Vibrio Infections in the Oyster *Crassostrea* *gigas* by Digital Gene Expression Analysis. *PLOS one*, 6: 8,

Li, Q., Wang, Q., Qi, M., Ge, J., and Cong, R. (2011). Development, characterization, and inheritance of 113 novel EST-SSR markers in the Pacific oyster (*Crassostrea* *gigas*). *Genes & Genomics*. 33: 3, 313-316.

Hedgecock, D., Gaffney, P.M., Guo, X., Shin, G., Gracey, A., Qi, H., Li, L., Zhang, G., and Samanta, M.P. (2011). *Gigas*np: Integrating Genetic, Physical And Cytogenetic Maps Of The Pacific Oyster *Crassostrea* *gigas*. *Journal of Shellfish Research*. 30: 2, 515-515.

Plough, L., and Hedgecock, D. (2011). Genotype by environment interaction affects genetic load in the Pacific Oyster, *Crassostrea* *Gigas*. *Journal of Shellfish Research*. 30: 2, 544-544.

Camara, M. D. (2011). Changes in molecular genetic variation at AFLP loci associated with naturalization and domestication of the Pacific oyster (*Crassostrea* *gigas*). *Aquatic Living Resources*. 24: 1, 35- 43.

Moehler, J., Wegner, K. M. Reise, K., and Jacobsen, S. (2011). Invasion genetics of Pacific oyster *Crassostrea* *gigas* shaped by aquaculture stocking practices. *Journal Of Sea Research*. 66: 3, 256-262.

Samain, J.-F. (2011). Review and perspectives of physiological mechanisms underlying genetically-based resistance of the Pacific oyster *Crassostrea* *gigas* to summer mortality. *Aquatic Living Resources*. 24: 3, 227-236.

Enriquez-Espinoza, TL., and Grijalva-Chon, JM. (2010). Variabilidad genética de *Crassostrea* *gigas* y *Crassostrea* *corteziensis* de un laboratorio de producción del noroeste de México. *Ciencias marinas*. 36: 4, 333-344.

Sauvage, C., Boudry, P., de Koning, D. -J., Haley, C. S., Heurtebise, S. and Lapegue, S. (2010). QTL for resistance to summer mortality and OsHV-1 load in the Pacific oyster (*Crassostrea* *gigas*). *Animal Genetics*. 41: 4, 390-399.

Degremont, L., Bedier, E., and Boudry, P. (2010). Summer mortality of hatchery-produced Pacific oyster spat (*Crassostrea* *gigas*). II. Response to selection for survival and its influence on growth and yield. *Aquaculture*. 299: 1-4, 21-29.

Degremont, L., Boudry, P., Ropert, M., Samain, J.-F., Bedier, E., and Soletchnik, P. (2010). Effects of age and environment on survival of summer mortality by two selected groups of the Pacific oyster *Crassostrea* *gigas*. *Aquaculture*. 299: 1-4, 44-50.

Sussarellu, R., Fabioux, C., Le Moullac, G., Fleury, E., and Moraga, D. (2010). Transcriptomic response of the Pacific oyster *Crassostrea* *gigas* to hypoxia. *Marine Genomics*. 3: 3-4, 133-143.

Huvet, A., Normand, J., Fleury, E., Quillien, V., Fabioux, C., and Boudry, P. ( 2010). Reproductive effort of Pacific oysters: A trait associated with susceptibility to summer mortality. *Aquaculture*. 304: 1-4, 95-99.

Meyer, E., and Manahan, D. T. (2010). Gene expression profiling of genetically determined growth variation in bivalve larvae (*Crassostrea* *gigas*). *Journal Of Experimental Biology*. 213: 5, 749-758.

Schmitt, P., Gueguen, Y., Desmarais, E., Bachere, E., and de Lorgeril, J. (2010). Molecular diversity of antimicrobial effectors in the oyster *Crassostrea* *gigas*. *BMC Evolutionary Biology*. 10, 23.

Hedrick, P.W., and Hedgecock, D. (2010). Sex Determination: Genetic models for oysters. *Journal of Heredity*. 101: 5, 602-611.
