# Supplementary material for: Genetic considerations for mollusk production in aquaculture: current state of knowledge
Source: Front Genet. 2014 Dec 10;5:435. doi: 10.3389/fgene.2014.00435 (PMC4261805; doi:10.3389/fgene.2014.00435)
Supplement: Supplementary file 3 [file DataSheet3.DOCX]

**Annex 3**

**References *Mytilus chilensis***

Order by publication date, newest to oldest

Larrain, A., Diaz, M., Lamas, NF., Uribe, C., and Araneda, C. (2014). Traceability of mussel (*Mytilus chilensis*) in southern Chile using microsatellite molecular markers and assignment algorithms. Exploratory survey. *Food Research International*. 62: 104-110.

Larrain, A., Diaz, M., Lamas, NF., Vargas, C., and Araneda, C. (2012). Genetic composition of Mytilus species in mussel populations from southern Chile. *Latin American Journal of Aquatic Research*. 40: 4, 1077-1084.

Nunez-Acuna, G., Tapia, FJ., Haye, PA., and Gallardo-Escarate, C. (2012). Gene expression analysis in *Mytilus chilensis* populations reveals local patterns associated with ocean environmental conditions. *Journal of Experimental Marine Biology and Ecology*. 420: 56-64.

Lagos, L., Uriarte, I., Yany, G., and Astorga, M. (2012). Effect of temperature on the culture of larvae of the bivalve *Mytilus chilensis* originated from broodstocks from different latitudes in a controlled environment. *Ciencias Marinas*. 38: 3, 543-550.

Borsa, P., Rolland, V., and Daguin-Thiebaut, C. (2012). Genetics and taxonomy of Chilean smooth-shelled mussels, Mytilus spp. (Bivalvia: Mytilidae). *Comptes Rendus Biologies*. 335: 1, 51-61.

Ouagajjou, Y., Presa, P., Astorga, M., and Perez, M. (2011). Microsatellites of *Mytilus chilensis:* a genomic print of its taxonomic status within *Mytilus sp*. *Journal of Shellfish Research*. 30: 2, 325-330.

Alcapan, AC., Nespolo, RF., and Toro, JE. (2007). Heritability of body size in the Chilean blue mussel *(Mytilus chilensis* Hupe 1854): effects of environment and ageing. *Aquaculture Research*. 38: 3, 313-320.

Krapivka, S., Toro, JE., Alcapan, AC., Astorga, M., Presa, P., Perez, M., and Guinez, R. (2007). Shell-shape variation along the latitudinal range of the Chilean blue mussel *Mytilus chilensis* (Hupe 1854). *Aquaculture Research*. 38:16, 1770-1777.

Toro, JE., Castro, GC., Ojeda, JA., and Vergara, AM. (2006). Allozymic variation and differentiation in the chilean blue mussel, *Mytilus chilensis*, along its natural distribution. *Genetics and Molecular Biology*. 29: 1, 174-179.

Toro, JE., Ojeda, JA., Vergara, AM., Castro, GC., and Alcapan, AC. (2005). Molecular characterization of the Chilean blue mussel (*Mytilus chilensis* Hupe 1854) demonstrates evidence for the occurrence of *Mytilus galloprovincialis* in southern Chile. *Journal of Shellfish Research*. 24: 4, 1117-1121.

Carcamo, C., Comesana, AS., Winkler, FM., and Sanjuan, A. (2005). Allozyme identification of mussels (Bivalvia: Mytilus) on the Pacific coast of South America. *Journal of Shellfish Research*. 24: 4, 1101-1115.

Toro, JE., Alcapan, AC., Ojeda, JA., and Vergara, AM. (2004). Selection response for growth rate (shell height and live weight) in the chilean blue mussel (*Mytilus chilensis* hupe 1854). *Journal of Shellfish Research*. 23: 3, 753-757.

Toro, JE., Ojeda, JA., and Vergara, AM. (2004). The genetic structure of Mytilus chilensis (Hupe 1854) populations along the Chilean coast based on RAPDs analysis. *Aquaculture Research*. 35: 15, 1466-1471.

Rego, I., Martinez, A., Gonzalez-Tizon, A., Vieites, J., Leira, F., and Mendez, J. (2002). PCR technique for identification of mussel species. *Journal of Agricultural and Food Chemistry*. 50: 7, 1780-1784.

Toro, JE., and Paredes, LI. (1996). Heritability estimates of larval shell length in the Chilean blue mussel *Mytilus chilensis*, under different food densities. *Aquatic Living Resources*. 9: 4, 347-350.
