# Supplementary material for: Genetic considerations for mollusk production in aquaculture: current state of knowledge
Source: Front Genet. 2014 Dec 10;5:435. doi: 10.3389/fgene.2014.00435 (PMC4261805; doi:10.3389/fgene.2014.00435)
Supplement: Supplementary file 4 [file DataSheet4.DOC]

**Annex 4.**

**References** *Tegillarca granosa*

Order by publication date, newest to oldest

Ni, G., Li, Q., Kong, L., and Yu, H. (2014). Comparative phylogeography in marginal seas of the northwestern Pacific. *Molecular Ecology*. 23: 3, 534-548.

Yingkajorn, M., Sermwitayawong, N., Palittapongarnpimp, P., Nishibuchi, M., Robins, WP., Mekalanos, JJ., and Vuddhakul, V. (2014). Vibrio parahaemolyticus and Its Specific Bacteriophages as an Indicator in Cockles (*Anadara granosa*) for the Risk of V-parahaemolyticus Infection in Southern Thailand. *Microbial Ecology*. 67: 4, 849-856.

Wang, YJ., Zeng, QG., and Xu, LN. (2013). Population structure of the blood clam (*Tegillarca granosa*) in China based on microsatellite markers. *Genetics and Molecular* *Research*. 12: 2, 892-900.

Ni, G., Li, Q., Kong, L., and Zheng, X. (2012). Phylogeography of the bivalve *Tegillarca granosa* in coastal China: implications for management and conservation. *Marine Ecology Progress Series*. 452: 119-130.

Liu, B., Teng, S-S., Shao, Y-Q., Chai, X-L., and Xiao, G-Q. (2012). Isolation and characterization of 39 novel polymorphic EST-SSR loci for the blood clam, *Tegillarca granosa*. *Conservation Genetics Resources.* 4: 2, 375-378.

Li, M., Zhu, L., Zhou, C-Y., Lin, L., Fan, Y-J., and Zhuang, Z-M. (2012). Development and Characterization of EST-SSR Markers from *Scapharca broughtonii* and Their Transferability in *Scapharca subcrenata* and *Tegillarca granosa*. *Molecules*. 17: 9, 10716-10723.

Feng, Y., Li, Q., Kong, L., and Zheng, X. (2011). COI-based DNA barcoding of Arcoida species (Bivalvia: Pteriomorphia) along the coast of China. *Molecular Ecology Resources.* 11: 3, 435-441.
