# Supplementary material for: Genetic considerations for mollusk production in aquaculture: current state of knowledge
Source: Front Genet. 2014 Dec 10;5:435. doi: 10.3389/fgene.2014.00435 (PMC4261805; doi:10.3389/fgene.2014.00435)
Supplement: Supplementary file 5 [file DataSheet5.DOC]

**Annex 5.**

**References** *Sinonovacula constricta*

Order by publication date, newest to oldest

Niu, D., Wang, L., Sun, F., Liu, Z., and Li, J. (2013). Development of Molecular Resources for an Intertidal Clam, *Sinonovacula constricta*, Using 454 Transcriptome Sequencing. *Plos One*. 8: 7.

Wang, L., Niu, D., and Li, J. (2013). Characterization of novel EST-derived SNP markers using 454 pyrosequencing in *Sinonovacula constricta*. *Conservation Genetics Resources*. 5: 1, 191-193.

Niu, D-H., Feng, B-B., Liu, D-B., Zhong, Y-M., Shen, H-D., and Li, J-L. (2012). Significant Genetic Differentiation among Ten Populations of the Razor Clam *Sinonovacula constricta* along the Coast of China Revealed by a Microsatellite Analysis. *Zoological Studies*. 51: 3, 406-414.

Jiang, Q., Li, Q., Yuan, Y., and Kong, L-F. (2010). Development and characterization of 14 polymorphic microsatellite loci in the razor clam (*Sinonovacula constricta*). *Conservation Genetics Resources*. 2: 81-83.

Niu, D-H., Li, J-L., and Liu, D-B. (2008). Polymorphic microsatellite loci for population studies of the razor clam, *Sinonovaculd constricta*. *Conservation Genetics*. 9: 5, 1393-1394.
